# Psychiatric symptoms in Long-COVID patients: a systematic review

**DOI:** 10.3389/fpsyt.2023.1138389

**Published:** 2023-06-21

**Authors:** Mattia Marchi, Pietro Grenzi, Valentina Serafini, Francesco Capoccia, Federico Rossi, Patrizia Marrino, Luca Pingani, Gian Maria Galeazzi, Silvia Ferrari

**Affiliations:** ^1^Department of Biomedical, Metabolic and Neural Sciences, University of Modena and Reggio Emilia, Modena, Italy; ^2^Department of Mental Health and Drug Abuse, Azienda USL-IRCCS di Reggio Emilia, Reggio Emilia, Italy

**Keywords:** Long-COVID syndrome, COVID-19, mental health, depression, anxiety, posttraumatic stress

## Abstract

**Objective:**

People who have been infected by COVID-19 showing persistent symptoms after 4 weeks from recovery are thought to suffer from Long-COVID syndrome (LC). There is uncertainty on the clinical manifestations of LC. We undertook a systematic review to summarize the available evidence about the main psychiatric manifestations of LC.

**Method:**

PubMed (Medline), Scopus, CINHAL, PsycINFO, and EMBASE were searched until May 2022. Studies reporting estimation of emerging psychiatric symptoms and/or psychiatric diagnoses among adult people with LC were included. Pooled prevalence for each psychiatric condition was calculated in absence of control groups to compare with.

**Results:**

Thirty-three reports were included in the final selection, corresponding to 282,711 participants with LC. After 4 weeks from COVID-19 infection recovery, participants reported the following psychiatric symptoms: depression, anxiety, post-traumatic symptoms (PTS), cognitive and sleeping disturbances (i.e., insomnia or hypersomnia). The most common psychiatric manifestation resulted to be sleep disturbances, followed by depression, PTS, anxiety, and cognitive impairment (i.e., attention and memory deficits). However, some estimates were affected by important outlier effect played by one study. If study weight was not considered, the most reported condition was anxiety.

**Conclusions:**

LC may have non-specific psychiatric manifestations. More research is needed to better define LC and to differentiate it from other post-infectious or post-hospitalization syndromes.

**Systematic review registration:**

PROSPERO (CRD42022299408).

## Introduction

Long-COVID syndrome (LC) is a condition that can affect people who have recovered from Coronavirus Disease 2019 (COVID-19). This term was introduced to indicate a set of disorders that persist or occur at from 4 weeks after the elimination of the SARS-CoV-2 virus from the body (1). The clinical features of LC are multifaceted; it has been posited that it can affect different organs and systems, causing somatic but also psychological manifestations that impact on quality of life (2).

For most people, mild or moderate COVID-19 lasts for about 2 weeks; in some cases, though, symptoms can persist or develop after healing. Furthermore, also in people with asymptomatic infections later health problems may develop (3–5).

Although progress has been made in the understanding of the clinical and epidemiological features, including the pathogenesis and complications of the acute phase of COVID-19, long-term consequences of the disease remain largely unclear (6).

Additionally, while neuropsychiatric symptoms that manifest acutely during infection, such as depression, post-traumatic symptoms [PTS], sleep and cognitive disturbances or anxiety, have received more attention, the medium- and long-term psychiatric outcomes in COVID-19 patients are still little known and understudied (7, 8).

In the available literature, there are highly heterogeneous research works on this topic, applying widely different sample sizes, inclusion and exclusion criteria, and duration of follow-up. In addition, patient assessment is mainly based on various assessment tools and questionnaires, self-administered in most cases, that do not provide a diagnosis of a condition with definite clinical significance.

Therefore, understanding the medium and long-term impact of COVID-19 is still far from being complete, not only in the context of a multidisciplinary approach, but even more so when focusing on specific areas such as mental health (1).

We undertook this systematic review to summarize the available evidence about the main psychiatric manifestations of LC. A better understanding of the epidemiology of psychopathological manifestations among LC patients is crucial to develop prevention and early interventions.

## Methods

This systematic review was performed according to the Preferred Reporting Items for Systematic Reviews and Meta-Analyses (PRISMA) guidelines. The protocol of this systematic review was registered with PROSPERO (CRD42022299408).

### Data sources and search strategy

We searched the PubMed (Medline), Scopus, CINHAL, PsycINFO, and EMBASE databases until May 2022, using the strategy outlined in the Supplementary Table 1 of the Appendix. In addition, the list of references of the included studies and of other reviews on related topics was screened to identify any other possible study deserving inclusion, and inadvertently missed during the initial literature search. No restrictions regarding language of publication or publication date were set.

### Eligibility criteria

We included experimental and observational studies reporting estimation of rates of emerging psychiatric symptoms and/or psychiatric diagnosis among adult people (i.e., ≥18 years old) with LC, without any restriction on other medical comorbidities or setting of enrolment. We excluded studies on participants already suffering from any psychiatric condition, studies assessing the presence of psychiatric symptoms before 4 weeks from COVID-19 recovery, and previous reviews, case-reports, case-series, editorial, and letters to the editor. We only included studies published in peer-reviewed journals, excluding conference abstracts and dissertations. If data from the same sample were published in multiple works, we considered only that study reporting more exhaustive information. Sample overlap was ruled out through a careful check of the registration codes as well as the place and year(s) of sampling.

Where available, outcome data from participants with other inflammatory or infectious diseases, including COVID-19 but without LC, were used as control group.

### Terms and definitions

LC was defined as either the presence or the persistence of any symptom that was not present before the infection after 4 weeks from the COVID-19 recovery. Infection from SARS-CoV-2 and recovery from the infection were defined according to the result of the real-time PCR on nasopharyngeal swab sample, or of broncho-alveolar lavage.

Psychiatric symptoms were collected from self-reporting or from validated psychometric tools. Where a psychiatric diagnosis was reported, it had to be defined according to standard operational diagnostic criteria (the Diagnostic and Statistical Manual of Mental Disorders [DSM] or the International Classification of Diseases [ICD]).

### Data collection and extraction

Four Reviewers (P.G., V.S., F.R., and F.C.) working independently preliminarily reviewed titles and abstracts of retrieved articles. The initial screening was followed by the analysis of full texts to check compliance with inclusion/exclusion criteria. All disagreements were discussed until consensus, and if consensus was not possible, another member of the team was consulted (M.M.). A standardized form was used for data extraction. Information concerning the year of publication, country, setting, characteristics of study participants (sample size, age, percentages of men and women), LC status, and the presence of psychiatric conditions in the LC groups (and, where available, in the control group) were collected by two authors (P.G. and V.S.) independently. Extraction sheets for each study were cross-checked for consistency and any disagreement was resolved by discussion within the research group.

### Statistical analyses

Where possible (i.e., there were at least two studies providing outcome data for LC and controls), quantitative data among studies were summarized using random effects meta-analysis (9). To summarize continuous outcome data (i.e., the scores on a psychometric tool), the pooled Hedges' g standardized mean differences (SMDs) and the corresponding 95% confidence intervals (CIs) were applied, while pooled odds ratios (ORs) and the corresponding 95% CIs (10) were used to report on dichotomous outcome data (i.e., presence/absence of psychiatric diagnosis or psychiatric symptoms).

If meta-analysis was not possible, we calculated the pooled prevalence of psychiatric symptoms and/or psychiatric diagnosis among LC patients. These estimates consisted in weighted-mean prevalence, raw mean prevalence, and median prevalence, with the relative lower and upper ranges across the studies included in the final selection.

The analyses were performed in R (11). Statistical tests were 2-sided and used a significance threshold of *p* < 0.05.

### Risk of bias assessment and the GRADE

Bias risk in the included studies was independently assessed by three reviewers (P.G., V.S., and F.R.), using the Cochrane risk of bias tool (12). All disagreements were discussed until consensus, and if necessary, another member of the team was consulted (M.M.). Each item on the risk of bias assessment was scored as high, low, or unclear, and the GRADE tool was used to assess the overall certainty of evidence (13). Further information is available in the Supplementary material.

## Results

### Study characteristics

**Figure 1:**
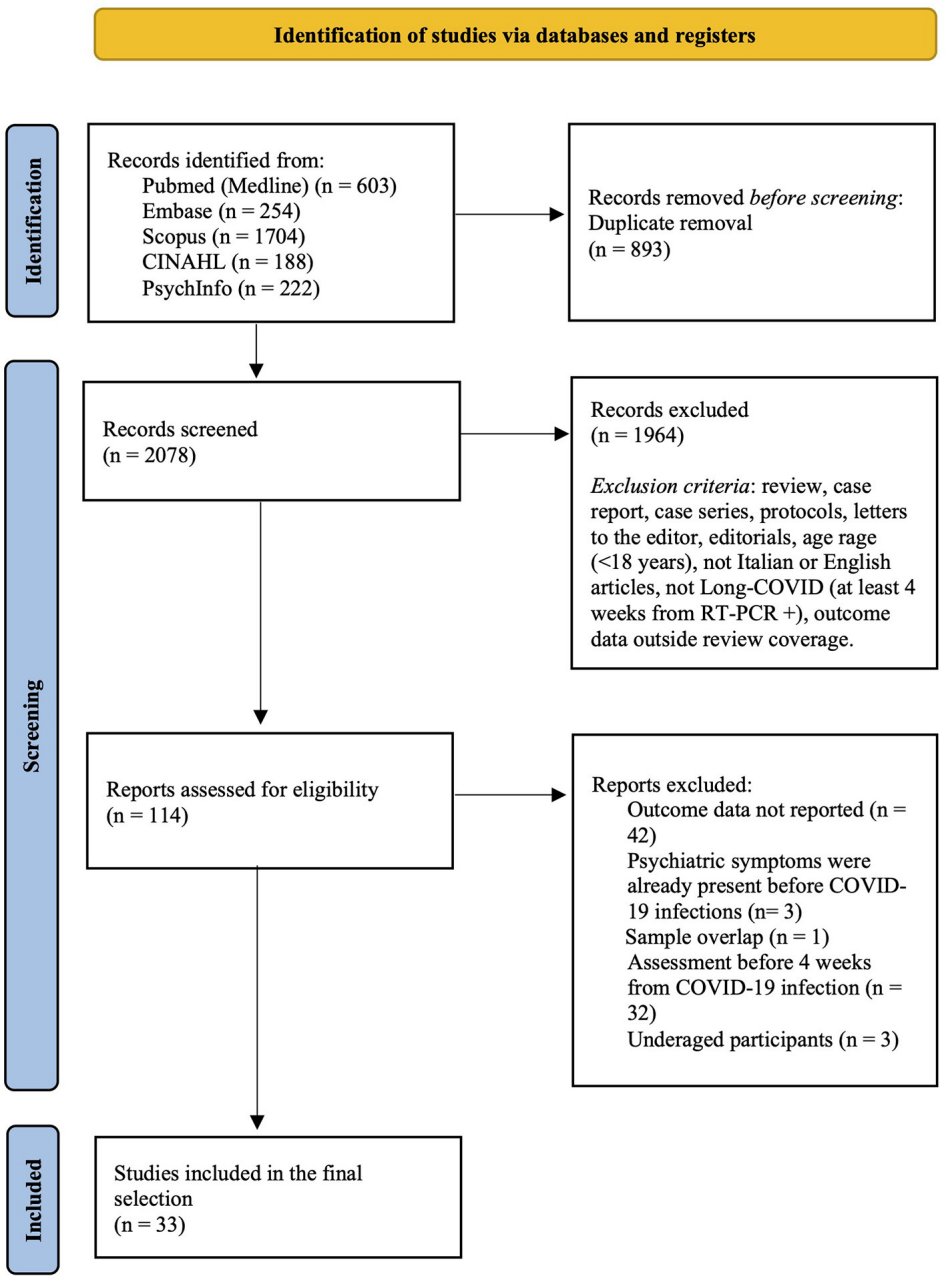
Preferred reporting items for systematic reviews and meta-analyses (PRISMA) flow diagram.

As shown in Figure 1, from 2078 records screened on title and abstract, 114 full texts were analyzed. The review process led to the selection of 33 studies (3, 4, 6–8, 14–41). These studies, referring to 33 different samples and involving a total of 282,711 LC participants, were included in the final selection and quantitative synthesis.

On an average, across the studies, 48% of participants were females (range 23.1–100%). The mean age of participants was 53.2 years (range 33.7–73.2). The selected studies were conducted in 13 countries: US (*n* = 6; 18.2%); Italy (*n* = 5; 15.2%); Egypt, France, Netherlands, Spain, UK (each *n* = 3; 9.1%); India (*n* = 2; 6.1%); Austria, China, Germany, Iran, Mexico (each *n* = 1; 3.0%).

All the studies were published in the last 2 years: 2021 (*n* = 31; 93.9%); 2022 (*n* = 2; 6.1%).

With respect to the outcomes reported, only 2 studies (6.1%) provided data about psychiatric diagnosis: one study assessed depressive and anxiety disorder (GAD) through a clinical interview, the other study used retrospective screening of the electronic clinical records to investigate prevalence of anxiety and depression, and cognitive impairment, according to the ICD-10 system. The remaining studies (*n* = 31, 93.9%) used self-reporting or other psychometric tools to measure the level of: depression or anxiety (*n* = 26, 78.8%), cognitive impairment (*n* = 16, 48.5%), PTS (*n* = 13, 39.4%), and sleep disturbances (*n* = 18, 54.5%). These studies applied dichotomization into positive/negative at the psychometric assessment based on the scales' cut-off for clinical significance, and the estimated prevalence for each study was calculated as the number of participants with score above the cut-off divided to the total number of participants assessed.

Notably, none of the studies included in the final selection applied a control group without LC. Concerning the severity of COVID-19 infection, 15 studies (45.5%) included patients hospitalized due to COVID-19 infection, 5 studies (15.2%) were performed on patients who had mild infection not requiring hospitalization, and 11 studies (33.3%) included both hospitalized and other managed outpatients. Information about infection severity was missing in 2 studies (6.1%).

All studies characteristics are summarized in Table 1.

**Table 1:** Characteristics of the included studies.

**Author, year**	**Country**	**Study design**	**Females %**	**Mean age**	**N**	**Severity of COVID-19 infection**	**P depression (measure)**	**P Anxiety (measure)**	**P Cognitive impairment (measure)**	**P PTS (measure)**	**P Sleep disturbances (measure)**
Ahmed et al. (14)	Egypt	Cohort	54%	46.5	182	Both hospitalized and outpatients	0.374 (SCL-90)	0.619 (SCL-90)	NR	0.286 (PCL-5)	0.648 (PSQI)
Aly and Saber (15)	Egypt	Cross-sectional	100%	73.2	115	NR	NR	NR	0.252 (self-reported)	NR	0.243 (self-reported)
Aranda et al. (16)	Spain	Cohort	30%	64	113	Hospitalized	0.301 (BDI)	0.487 (STAI)	NR	0.788 (IES-R)	0.292 (NR)
Bai et al. (17)	Italy	Cohort	36%	57	377	Both hospitalized and outpatients	0.106 (HADS)	0.188 (HADS)	0.202 (NR)	0.225 (IES-R)	NR
Boesl et al. (18)	Italy	Cohort	67%	45.8	100	Mild	0.615 (BDI)	NR	0.306 (MoCA)	NR	0.337 (ESS)
De Graaf et al. (3)	Netherlands	Cohort	37%	60.8	81	Both hospitalized and outpatients	0.123 (PHQ-9)	0.037 (GAD-7)	0.160 (CFQ-25)	0.062 (PCL-5)	NR
Evans et al. (4)	UK	Cohort	36%	57.9	1077	Hospitalized	0.262 (PHQ-9)	0.235 (GAD-7)	0.139 (NR)	0.117 (PCL-5)	NR
Frontera et al. (19)	US	Case-control	35%	68.5	280	Hospitalized	0.254 (NeuroQoL)	0.511 (NeuroQoL)	0.473 (MoCA)	NR	0.375 (NeuroQoL)
Ganesh et al. (20)	US	Cohort	61%	44	817	Both hospitalized and outpatients	NR	NR	NR	NR	0.200 (PROMIS)
Garjani et al. (21)	UK	Cohort	82%	50	165	Mild	0.504 (NR)^†^	NR	NR	NR	NR
Gonzàlez-Hermosillo et al. (22)	Mexico	Cohort	35%	51	130	Hospitalized	0.354 (self-reported)	0.392 (self-reported)	0.454 (self-reported)	NR	0.454 (self-reported)
Gouraud et al. (23)	France	Cohort	29%	60	100	Hospitalized	0.220 (HADS)	0.310 (HADS)	NR	NR	NR
Graham et al. (24)	US	Case-control	66%	43.7	50	Mild	0.400 (NR)^†^	NR	0.820 (PROMIS)	NR	0.360 (PROMIS)
Gramaglia et al. (6)	Italy	Cohort	40%	61	238	Both hospitalized and outpatients	0.294 (MINI)	0.328 (MINI)	NR	0.429 (NR)	NR
Horwitz et al. (7)	US	Cohort	40%	62	126	Hospitalized	NR	NR	0.413 (PROMIS)	NR	0.349 (PROMIS)
Huang et al. (8)	China	Cohort	48%	57	1733	Hospitalized	0.227 (interview)^†^	NR	NR	NR	0.264 (interview)
Imran et al. (25)	India	Cross-sectional	33%	44.5	103	Hospitalized	0.126 (PHQ-9)	0.214 (GAD-7)	NR	0.087 (PCL-5)	NR
Lemhofer et al. (26)	Germany	Cross-sectional	59%	49.8	365	Mild	NR	0.249 (NR)	NR	NR	0.301 (NR)
Lombardo et al. (27)	Italy	Cohort	54%	53	303	Both hospitalized and outpatients	NR	NR	0.363 (semi-structured interview)	NR	0.465 (semi-structured interview)
Mendez et al. (28)	Spain	Cohort	41%	57	179	Hospitalized	0.268 (PHQ-9)	0.296 (GAD-7)	0.184 (NR)	0.251 (DTS)	NR
Morin et al. (37)	France	Cohort	42%	60.9	478	Hospitalized	0.206 (BDI)	0.314 (HADS)	0.384 (MoCA, D2-R, Q3PC)	0.142 (PCL-5)	0.536 (ISI)
Naik et al. (29)	India	Cohort	31%	41.6	272	Both hospitalized and outpatients	0.022 (interview)	0.029 (interview)	NR	NR	0.063 (interview)
Rass et al. (30)	Austria	Cohort	39%	55	90	Both hospitalized and outpatients	0.121 (HADS)	0.222 (HADS)	NR	0.100 (PCL-5)	NR
Romero-Duarte et al. (31)	Spain	Cohort	46%	63	794	Hospitalized	0.044 (NR)	NR	NR	NR	0.049 (NR)
Scherlinger et al. (32)	France	Case-control	66%	40	30	Mild	0.100 (Psychological interview)	0.267 (Psychological interview)	NR	0.300 (PCL-5)	NR
Simani et al. (33)	Iran	Cohort	33%	54.62	120	Hospitalized	NR	NR	NR	0.058 (PCL-5)	NR
Sykes et al., (34)	UK	Cohort	34%		134	Hospitalized	0.396 (self-reported)	0.478 (self-reported)	0.097 (self-reported)	NR	0.351 (self-reported)
Taquet et al. (35)	Netherlands	Cohort	58%	39.4	273618	Both hospitalized and outpatients	0.155 (ICD-10)^†^	NR	0.040 (ICD-10)	NR	NR
Tawfik (36)	Egypt	Cohort	58%	33.7	120	Both hospitalized and outpatients	NR	NR	0.008 (NR)	NR	0.042 (NR)
Van den Borst et al. (38)	Netherlands	Cohort	40%	59	124	Both hospitalized and outpatients	0.117 (HADS)	0.100 (HADS)	NR	0.073 (PCL-5)	NR
Vanichkachorn et al. (39)	US	Cohort	68%	45.7	100	NR	NR	NR	0.005 (NR)	NR	0.003 (NR)
Vannorsdall et al. (40)	US	Cohort	59%	54.5	82	Hospitalized	0.902 (PHQ-9)	0.646 (GAD-7)	0.805 (QDRS)	0.25 (IES-R)	NR
Vassalini et al. (41)	Italy	Cohort	46%	57	115	Hospitalized	0.148 (PHQ-9)	NR	NR	NR	NR

P, prevalence; PTS, post-traumatic symptoms; NR, not-reported; SCL-90, symptom checklist 90; PCL-5, PTSD checklist for DSM-5; PSQI, Pittsburgh sleep quality index; BDI, Beck depression inventory; STAI, state-trait anxiety inventory; IES-R, impact of event scale—revised; HADS, hospital anxiety and depression scale; MoCA, Montreal cognitive assessment; ESS, Epworth sleepiness scale; PHQ-9, patient health questionnaire-9; GAD-7, general anxiety disorder-7; CFQ-25, cognitive failure questionnaire-25; UK, United Kingdom; US, United States of America; NeuroQoL, health-related quality of life for clinical research in neurology; PROMIS, patient-reported outcomes measurement information system; MINI, mini-international neuropsychiatric interview; DTS, Davidson trauma scale; D2-R, D2 test of attention—revised; Q3PC, Q3PC cognitive screening questionnaire; ISI, insomnia severity index; ICD-10, international classification of diseases 10th revision; QDRS, quick dementia rating scale.

^†^depression and anxiety aggregated prevalence.

### Prevalence of psychiatric symptoms across the studies

Table 2 summarize the pooled prevalence estimates for each psychiatric symptom across the studies included in this review, and the population prevalence worldwide. Notably, prevalence of depression, anxiety, cognitive impairment, PTS, and sleep disturbances resulted much higher among LC patients than in the general population (42–46).

**Table 2:** Pooled prevalence of psychiatric symptoms across the included studies and worldwide prevalence.

**Outcome**	**Weighted mean P**	**Mean P (min; max)**	**Median P**	**N studies (LC participants)**	**World P**
Depression	0.212	0.254 (0.022; 0.902)	0.220	21 (5,079)	0.038
Anxiety	0.158	0.313 (0.029; 0.646)	0.296	23 (28,001)	0.040
Cognitive impairment	0.042	0.269 (0.005; 0.820)	0.227	16 (277,268)	0.011
PTS	0.192	0.218 (0.058; 0.788)	0.130	13 (3,162)	0.036
Sleeping disturbances	0.270	0.296 (0.003; 0.648)	0.319	18 (6,212)	0.038

P, prevalence; min, minimum; max, maximum; LC, Long-COVID syndrome; NA, not available; PTS, post-traumatic symptoms.

### Prevalence of depression

**Figure 2:**
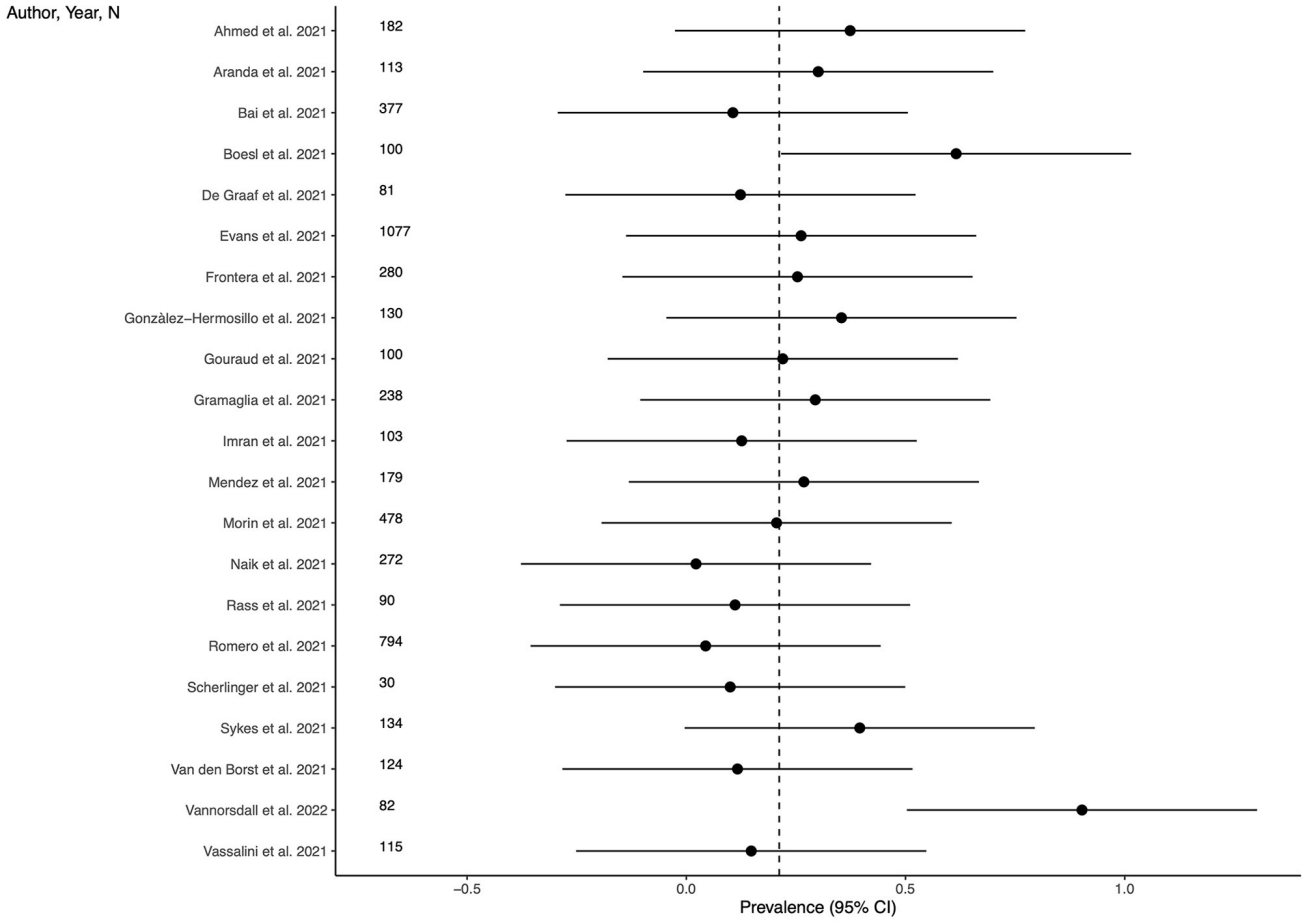
Prevalence of depression across the studies and weighted mean prevalence. The vertical dotted line represents the weighted mean prevalence.

Twenty-one studies (63.3%) provided outcome data for depression among LC patients. The weighted mean prevalence across the studies was 0.212, that is quite similar to the unweighted mean and median prevalence (0.254 [range: 0.022–0.902] and 0.220, respectively), consistent with not significant outlier effect played by any of the study in the pooled estimate. Figure 2 shows comparison of the depression prevalence estimates across the studies, and the weighted mean prevalence.

### Prevalence of anxiety

**Figure 3:**
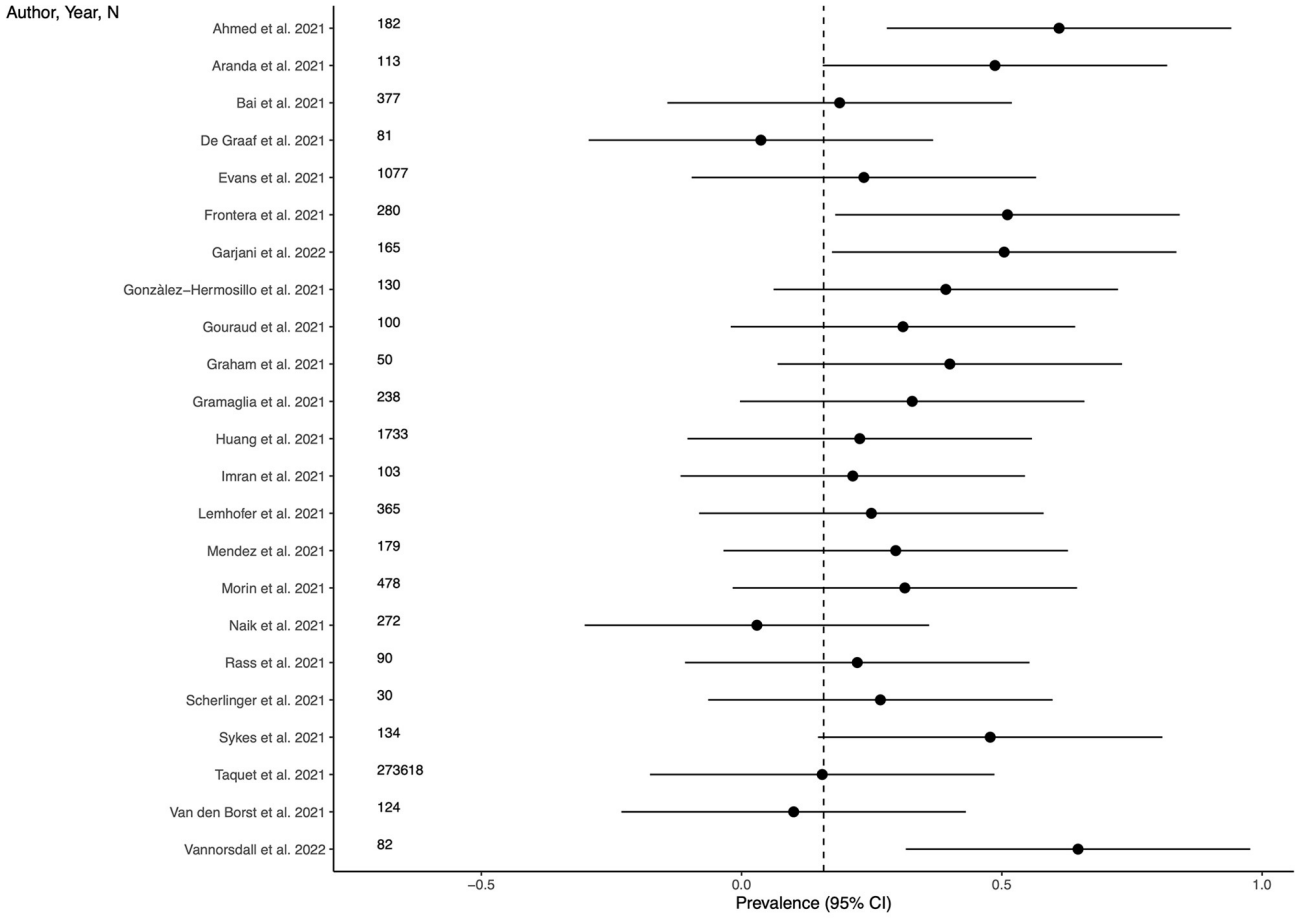
Prevalence of anxiety across the studies and weighted mean prevalence. The vertical dotted line represents the weighted mean prevalence.

Twenty-three studies (69.7%) provided outcome data for anxiety among LC patients. The weighted mean prevalence across the studies was 0.158 and was markedly influenced by the study from Taquet et al. (35) with a far larger sample size. Unweighted mean and median prevalence were 0.313 (range: 0.029–0.646) and 0.296, respectively. Figure 3 shows comparison of the anxiety prevalence estimates across the studies, and the weighted mean prevalence.

### Prevalence of cognitive impairment

**Figure 4:**
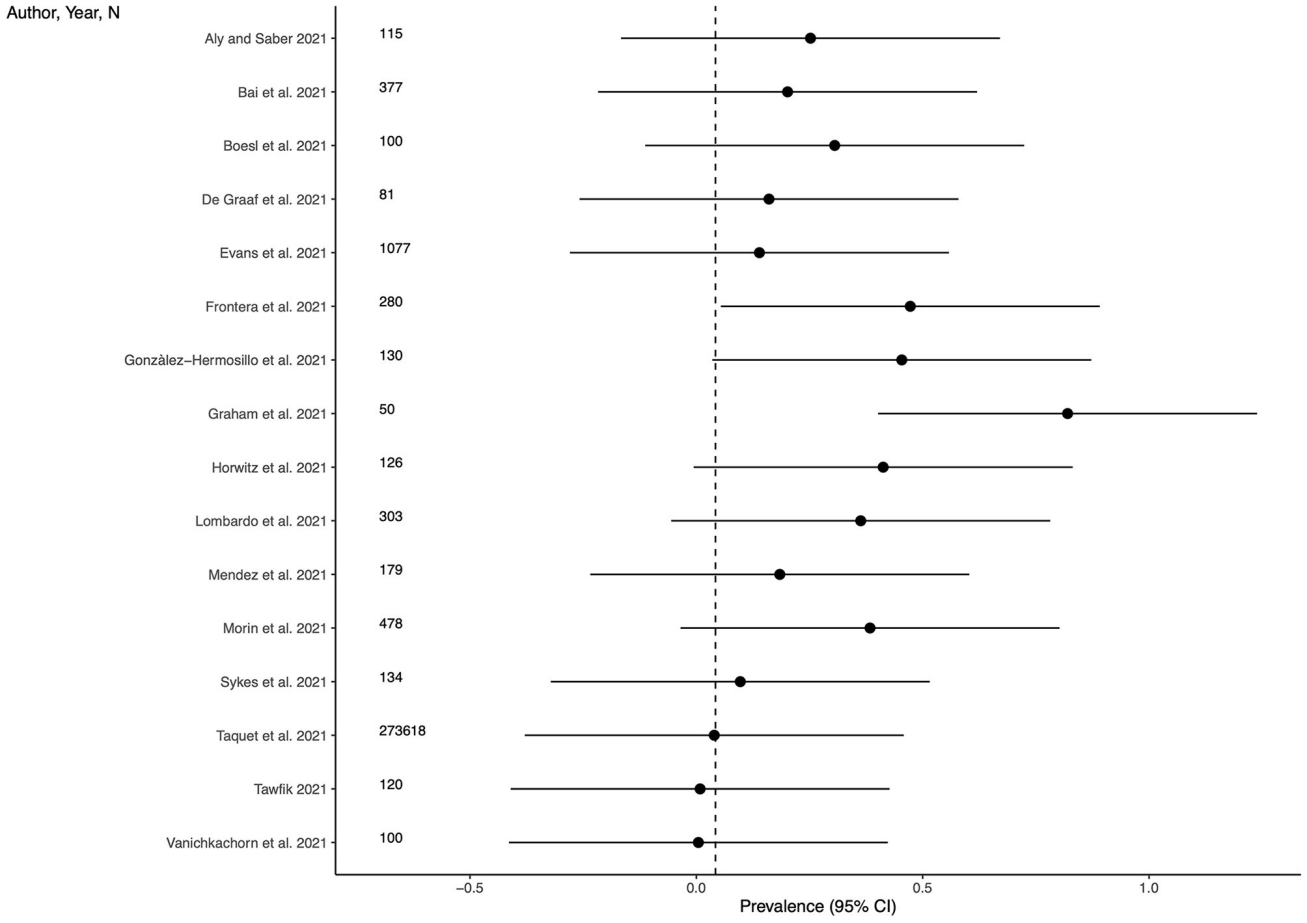
Prevalence of cognitive impairment across the studies and weighted mean prevalence. The vertical dotted line represents the weighted mean prevalence.

Sixteen studies (48.5%) provided outcome data for cognitive impairment among LC patients. The weighted mean prevalence across the studies was 0.042 and, again, was markedly influenced by the study from Taquet et al. (35) with the largest sample size and providing among the three lowest estimates of anxiety prevalence. Unweighted mean and median prevalence were 0.269 (range: 0.005–0.820) and 0.227, respectively. Figure 4 shows comparison of the cognitive impairment prevalence estimates across the studies, and the weighted mean prevalence.

### Prevalence of PTS

**Figure 5:**
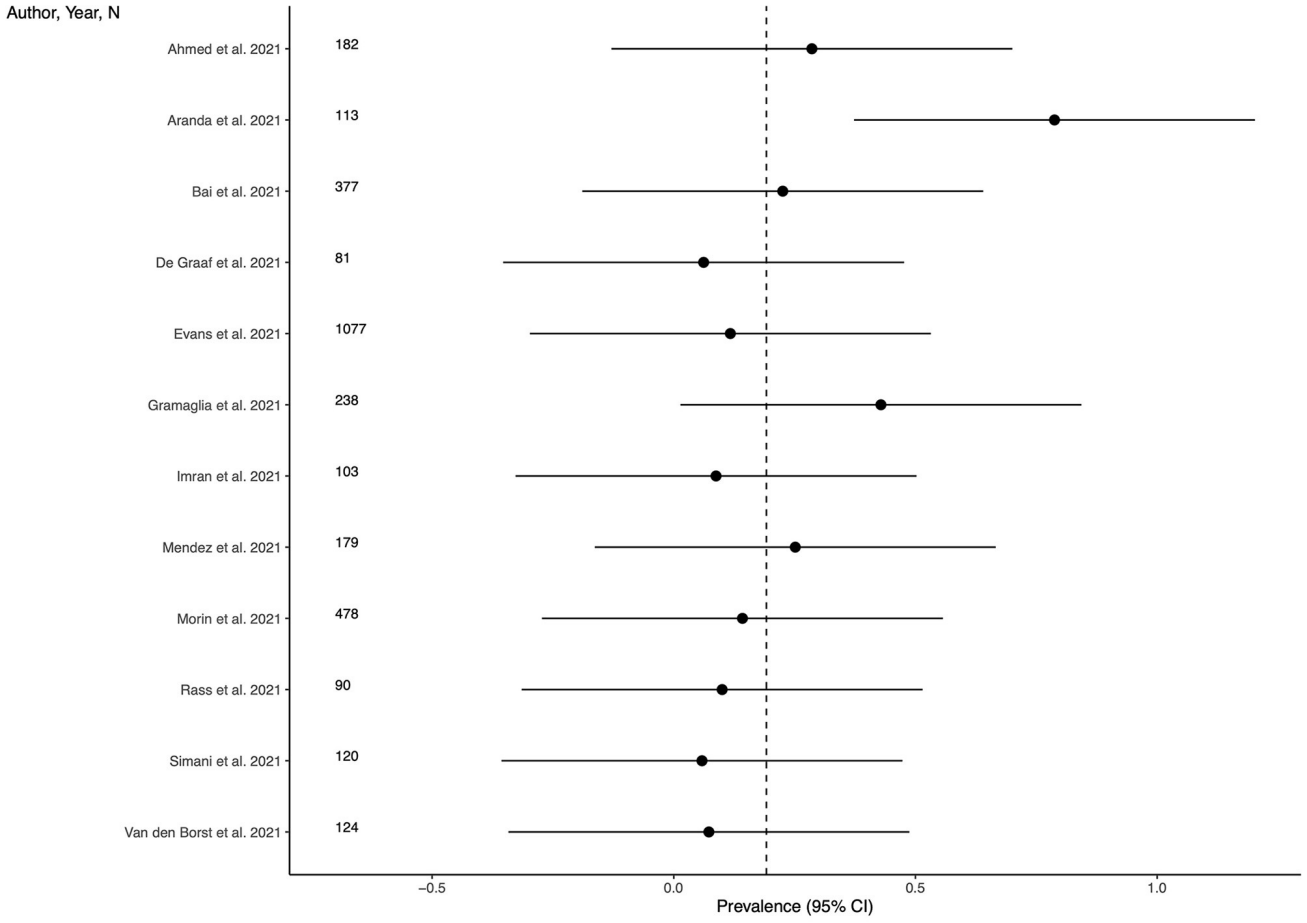
Prevalence of post-traumatic symptoms across the studies and weighted mean prevalence. The vertical dotted line represents the weighted mean prevalence.

Thirteen studies (39.4%) provided outcome data for PTS among LC patients. The weighted mean prevalence across the studies was 0.192. Unweighted mean and median prevalence were 0.218 (range: 0.058–0.788) and 0.130, respectively. Figure 5 shows comparison of the PTS prevalence estimates across the studies, and the weighted mean prevalence.

### Prevalence of sleep disturbances

**Figure 6:**
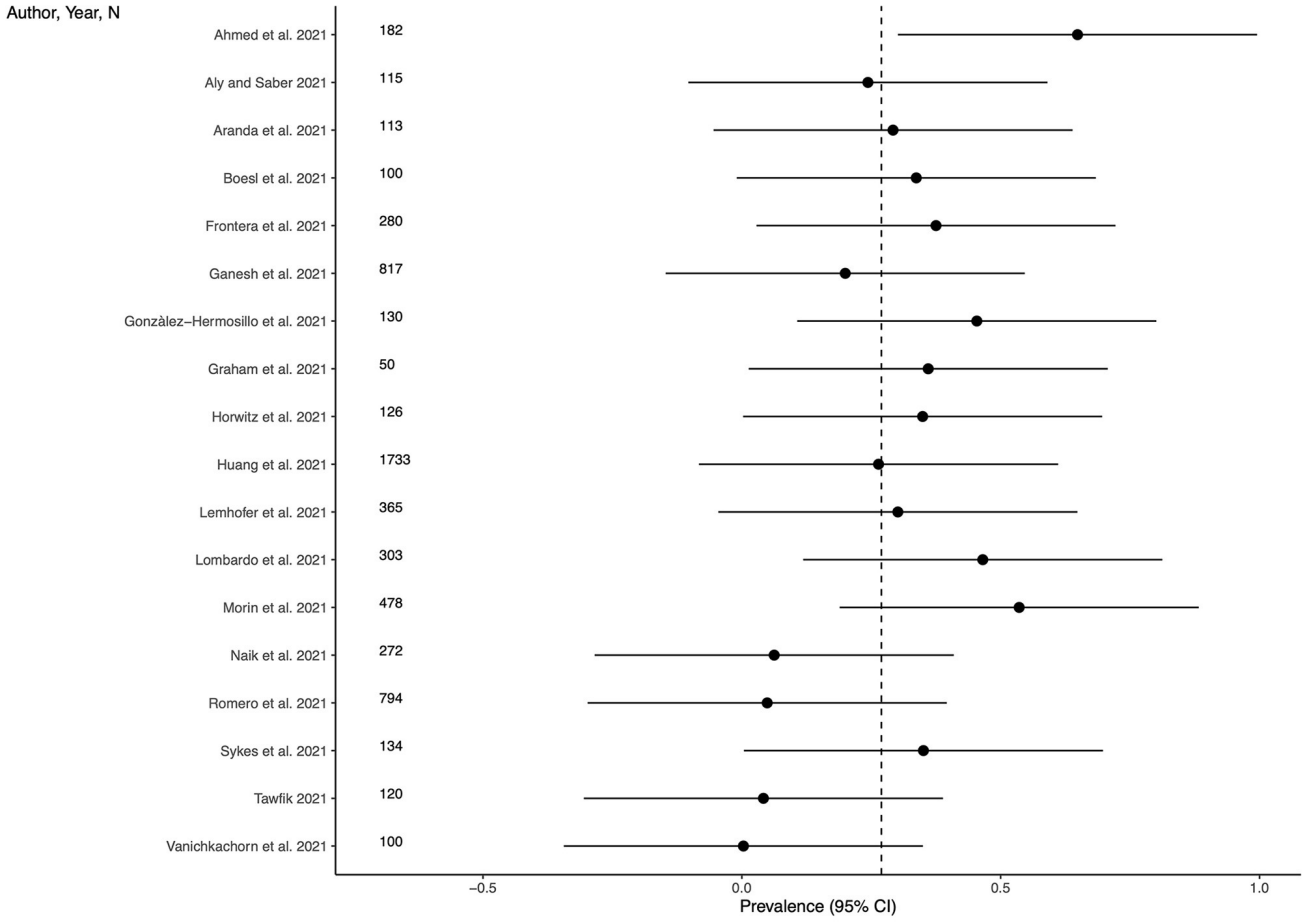
Prevalence of sleep disturbances across the studies and weighted mean prevalence. The vertical dotted line represents the weighted mean prevalence.

Eighteen studies (54.5%) provided outcome data for sleep disturbances among LC patients. The weighted mean prevalence across the studies was 0.270. Unweighted mean and median prevalence were 0.296 (range: 0.003–0.648) and 0.319, respectively. Figure 6 shows comparison of the sleep disturbances prevalence estimates across the studies, and the weighted mean prevalence.

### Psychiatric symptoms by COVID-19 infection severity

We examined the potential association between the severity of COVID-19 infection and the occurrence of psychiatric symptoms. For this purpose, we considered hospitalization as a proxy for severe infection, and outpatient management as indicator of mild infection. As shown in Table 3, the comparison of the severe, mild, and both mild and severe groups in terms of average prevalence of psychiatric symptoms did not find any statistically significant difference, suggesting that the severity of the infection is not related to the development of later psychiatric symptoms. Further analysis was conducted to compare only mild and severe patients, with the severe group consisting of studies that included at least one hospitalized patient. This analysis confirmed that there were no statistically significant differences in depression, anxiety, PTS, and sleep disturbances, but found inverse association between the severity of the infection and cognitive complaints (*p* = 0.048).

**Table 3:** Prevalence of psychiatric symptoms by severity of the COVID-19 infection.

	**Mean P (SD) mild**	**Mean P (SD) severe**	**Mean P (SD) both**	***p*-value**
N	5	15	11	
Depression	0.36 (0.36)	0.29 (0.22)	0.16 (0.12)	0.339
Anxiety	0.36 (0.12)	0.37 (0.14)	0.21 (0.19)	0.089
Cognitive impairment	0.56 (0.36)	0.31 (0.16)	0.15 (0.14)	0.061
PTS	NA	0.24 (0.28)	0.20 (0.15)	0.731
Sleeping disturbances	0.33 (0.03)	0.33 (0.14)	0.28 (0.26)	0.879

P, prevalence; SD, standard deviation; N, number; PTS, post-traumatic symptoms; NA, not available.

p-value are based on multiple groups analysis of variance (ANOVA).

### Risk of bias and GRADE

A detailed summary on the risk of bias in all 33 trials has been reported in the Appendix (see Supplementary Figures 1, 2), along with an assessment of the quality of the evidence (see Supplementary Table 2). In the GRADE system, the evidence from observational studies is initially set to low, there are then criteria that can be used either to downgrade or upgrade (see further information in the Appendix). The quality of the evidence is rated very low with serious threats related to the risk of bias and inconsistency.

## Discussion

This study set out to investigate the prevalence of psychiatric symptoms among LC patients. We found that the symptoms mostly associated with LC were depression, anxiety, cognitive and sleep disturbances, and PTS. The prevalence of these symptoms among LC patients is remarkably higher than that in the general population.

However, it is necessary to underline that, among the studies included in the final selection, there was one study (35) that had a sample size accounting for around 96% of the total number of participants included in this review. That study provided estimates for anxiety and cognitive impairments and the weighted average for these outcomes falls exactly on the value of the prevalence estimated by Taquet et al. (35) by looking at the forest plots of anxiety and cognitive impairment, it can be easily observed that only three studies for anxiety and two studies for cognitive impairment provided estimates smaller than Taquet et al. (35) supporting its influence on the pooled prevalence. Accordingly, for these two outcomes, the raw mean resulted higher than the weighted mean, because the former is not affected by the differences in the sample size across the studies.

Nevertheless, the relatively high pooled prevalence of psychiatric symptoms among LC patients requires a better understanding. Our analyses did not find a significant association between the severity of COVID-19 infection and psychiatric symptoms, except for a potential inverse association with symptoms of cognitive impairment. These findings align with the conclusions drawn from most of the reports included in our systematic review, which examined the relationship between infection severity and psychiatric symptoms (6, 17, 20, 23, 27, 34, 40). Notably, only one study reported an increased risk of depression and anxiety among individuals with the most severe form of infection (8). However, it is important to acknowledge that the confidence in the results is limited by the comparatively small representation of patients with mild infection, leading to low statistical power.

It should be noted that research in the pre-COVID-19 era observed that survivors after intensive care (IC) are at greater risk of developing long-term mental disorders (47). Particularly, anxiety, depression, and PTSD would have occurred in half of this sample of UK patients discharged from IC. Psychiatric symptoms of the post-IC syndrome fall into three broad categories: physical, cognitive, and psychological deterioration. Symptoms of physical deterioration include fatigue and insomnia, while cognitive and psychiatric symptoms include anxiety, depression, memory impairment, and PTSD. Therefore, there seems to be a significant overlap in the experience of some of the LC patients analyzed in this review with post-IC syndrome.

A considerable amount of COVID-related research also focused on the effect on mental health of public health measures (such as quarantine, lock-down, social isolation and other limitations to personal freedom), finding an association with symptoms of depression, anxiety, loneliness, psychosocial distress, and persisting post-traumatic arousal (48, 49). Therefore, another possible explanation for the higher prevalence of psychiatric symptoms among LC patients may be more a consequence of the imposed quarantine and other restrictions in terms of anxiety, fear, anger, and other negative emotions, regardless of specific aspects of the COVID-19 infection such as neuro- or systemic inflammation. Even if the quarantine imposed by a local health authority has not been directly associated with any psychological outcomes (50), it was suggested that belonging to a publicly recognized COVID-19 risk group/community would be associated with increased anxiety, depressive symptoms, self-concern, fear, increased psychosocial distress, and decreased life satisfaction. In addition, loneliness and isolation have been associated with an increased risk for various mental disorders (as well as for various somatic diseases). In the context of the COVID-19 pandemic, loneliness has been found to be predictive of depressive and anxious symptoms during the lockdown measures (51, 52).

COVID-19 is in fact only the most recent of many other infectious diseases that have been associated with chronic sequelae after recovering from the acute phase of infection (53): similarly as with LC, the underlying pathophysiological and etiological mechanisms are far from being clearly understood. The review by Choutka et al. (53) investigated the common characteristics between LC and other chronic infectious syndromes, finding higher prevalence of the following symptoms: intolerance to physical effort, neurocognitive and sensory impairment, persistent flu-like symptoms, disturbed sleep, myalgias, and arthralgias. The greatest analogies are with the post-acute effects described in the SARS epidemic in 2002–2004 (54).

All these elements are probably interrelated with each other and influential in the experience of LC patients.

### Limitations

The results of this review should be interpreted considering its limitations. First, the lack of a control group made difficult to draw considerations on the risk of psychiatric symptoms among LC patients, reducing considerably generalizability and reliability of our findings. This translated also in the impossibility to meta-analyze the results of the selected studies to detail the risk of psychiatric symptoms in patients who have had COVID-19. Second, most of the included studies reported measures of psychiatric symptoms instead of assessing psychiatric diagnoses, with a risk for diagnostic overestimation: this was partially attenuated by including only studies applying validated psychometric tools or clinical interviews. Third, there were a marked outlier effect played by one study (35), which implemented a sample size accounting for more than 90% of the total sample size. Even though that study resulted at low risk of bias in the assessment, it may have impacted on the pooled prevalence estimate of the outcomes reported in that study. Fourth, the condition of LC has been assessed only through a temporal criterion, that was 4 weeks after recovery from the infection. The lack of a more comprehensive definition of LC may have increase the heterogeneity in the estimates. Finally, the risk of bias was rated high or unclear in many studies, with serious threats related to the inconsistency in the estimates and to the assessment of confounders.

### Implications for research and clinical practice

The overlapping of some clinical features of LC in terms of signs and symptoms with other post-infectious syndromes and with the post-IC syndrome would suggest the involvement of shared pathophysiological pathways. The perspective of identifying a unified etiological model would lead the way toward the implementation of diagnostic markers and tailored treatments (53). At present, however, our understanding of the underlying pathophysiological mechanisms and etiological factors is poor, though promising studies are being conducted (55–57). For example, a recent review advanced the hypothesis that perivascular inflammation serves as the critical pathogenetic factor for LC neuropsychiatric manifestations (58). Indeed, SARS-CoV-2 and other viruses (such as retrovirus) showed the ability to activate brain mast cells and microglia resulting in the release of inflammatory, neurotoxic, and vasoactive mediators impacting neuronal connectivity and signal transmission (59–61). Hopefully, that may also converge to a better definition of functional and psycho-somatic syndromes, such as fibromyalgia and chronic fatigue syndrome, for which the association with viral infections has been previously proposed (62–67).

More research is therefore needed, more clearly comparing different patient groups (e.g., LC patients that were admitted to ICU vs. other ICU patients with or without other infectious diseases; LC patients vs. patients remitting from other infectious diseases) and applying prospective designs, allowing causal considerations, and providing more epidemiological details. Also, qualitative studies investigating the subjective experience of people recovered from COVID-19 are being conducted (68): this approach may also contribute to the understanding of the psychological mechanism contributing to the onset of psychological symptoms. Such different research methods could converge on a better conceptualization and analysis of the symptoms associated with the LC syndrome, as well as supporting the construction of a better defined and unified nomenclature.

A better understanding of the LC psycho-pathophysiology is essential to provide and improve treatment. From a therapeutic point of view, in close relation both to the traumatic component of a part of the symptoms found in LC, and to the inflammatory component (initially exerted by the infection and then self-sustained), interventions aimed at reducing the inflammatory process and reducing the excessive activation of the sympathetic nervous system through a relaxation response may be useful. For example, models of intervention involving reconditioning and mindfulness may help patients suffering from LC (69). Future clinical trials on LC patients may be therefore warranted.

## Conclusions

People who have recovered from COVID-19 may experience more and persistent psychiatric symptoms. These include depression, anxiety, post-traumatic distress, cognitive and sleeping disturbances. However, there is marked heterogeneity in the literature about how these symptoms are investigated and differentiated from other post-infectious or post-hospitalization conditions. More research, particularly implementing control groups and prospective follow-up, are needed to better define psychopathology related or included into the LC syndrome.

## Data availability statement

The raw data supporting the conclusions of this article will be made available by the authors, without undue reservation.

## Author contributions

MM, PG, and VS: conceptualization and planning and interpretation of the results. PG, VS, FC, FR, and PM: acquisition. MM: analysis of the data. MM, PG, VS, FC, FR, and PM: drafting. SF, LP, and GG: critical revision of the manuscript. All authors approved the final submitted version of the manuscript.
