# Supplementary material for: Psychiatric symptoms in Long-COVID patients: a systematic review
Source: Front Psychiatry. 2023 Jun 21;14:1138389. doi: 10.3389/fpsyt.2023.1138389 (PMC10320160; doi:10.3389/fpsyt.2023.1138389)
Supplement: Supplementary file 1 [file Data_Sheet_1.docx]

**Supplementary Materials**

Supplementary Table 1: Search strategy and number of hits per database (current to 30^th^ May 2022).

| **Database** | **String** | **Number of records** |
| --- | --- | --- |
| PubMed (Medline) | ((("Psychiatry"[Mesh]) OR ("Mental Health"[Mesh])) OR ("Mental Disorders"[Mesh])) AND ((long covid[MeSH Terms]) OR ("post-acute COVID-19 syndrome" [Supplementary Concept])) | 603 |
| Scopus | ( ( TITLE-ABS-KEY ( mental AND disorder ) OR TITLE-ABS-KEY ( mental AND health ) OR TITLE-ABS-KEY ( psychiatry ) ) ) AND ( TITLE-ABS-KEY ( long AND covid ) ) | 1704 |
| EMBASE | ('mental health'/exp OR 'mental health' OR 'mental disease'/exp OR 'mental disease' OR 'psychiatry'/exp OR psychiatry) AND ('long covid'/exp OR 'long covid') | 254 |
| CINAHL | (long covid or chronic covid-19 or post covid or long haul covid) AND (( mental health or mental illness or mental disorder or psychiatric illness ) OR psychiatry) | 188 |
| PsycINFO | ((mental health or mental illness or mental disorder or psychiatric illness ) OR psychiatry) AND (long covid or chronic covid-19 or post covid or long haul covid) | 222 |

Supplementary Figure 2: Risk of bias graph: review authors' judgements about each risk of bias item presented as percentages across all included studies.

Supplementary Figure 3: Risk of bias summary: review authors' judgements about each risk of bias item for each included study.

Supplementary Table 2: GRADE evidence summary for each outcome

| **Certainty assessment** | | | | | | **N of participants** | **Effect** | | **Certainty** |
| --- | --- | --- | --- | --- | --- | --- | --- | --- | --- |
| **N of studies** | **Study design** | **Risk of bias** | **Inconsistency** | **Indirectness** | **Outcome** | **LC** | **Weighted P** | **Mean P** |  |
| 21 | Observational studies | Serious | Not serious | Not serious | Depression | 5079 | 0.212 | 0.254 | +???  VERY LOW |
| 23 | Observational studies | Serious | Serious | Not serious | Anxiety | 28001 | 0.158 | 0.313 | ????  VERY LOW |
| 16 | Observational studies | Serious | Serious | Not serious | Cognitive impairment | 277268 | 0.042 | 0.269 | ????  VERY LOW |
| 13 | Observational studies | Serious | Not serious | Not serious | PTS | 3162 | 0.192 | 0.218 | +???  VERY LOW |
| 18 | Observational studies | Serious | Not serious | Not serious | Sleep disturbances | 6212 | 0.270 | 0.296 | +???  VERY LOW |

Abbreviations: LC: Long-COVID Syndrome; P: prevalence.

*Explanation of reasons for downgrading/upgrading:*

We GRADEd each pooled estimate for each relevant outcome according to the following criteria:

a. ***Risk of Bias:*** *We downgraded this domain by one level when any of the sources of Risk of Bias were rated as “high” or every two rated as “unclear” of the studies included in the pooled estimate.*

b. ***Inconsistency:*** *We downgraded this domain by one level if the difference between weighted mean pooled prevalence and mean pooled prevalence was ≥10%.*

c. ***Indirectness:*** *We protected against indirectness in this review by ensuring all studies included in the analysis assessed psychiatric symptoms using clinical interview or other validated psychometric tools. We therefore did not downgrade this domain.*
